# Clinical Effect of Superior Capsular Reconstruction With Long Head of the Biceps Tendon Autograft: Biceps Tenotomy Versus Retention in Massive Rotator Cuff Tears

**DOI:** 10.1111/os.70278

**Published:** 2026-03-17

**Authors:** Jiani Chen, Yimeng Yang, Shurong Zhang, Yang Wu, Jiwu Chen, Shiyi Chen, Xiliang Shang

**Affiliations:** ^1^ Department of Sports Medicine, Huashan Hospital Fudan University Shanghai China; ^2^ Department of Sports Medicine, Shanghai General Hospital Shanghai Jiao Tong University Shanghai China

**Keywords:** arthroscopy, biceps tenotomy, long head of the biceps tendon, rotator cuff tears, superior capsular reconstruction

## Abstract

**Background:**

Massive rotator cuff tears (RCTs) often lead to superior migration and poor function. While superior capsular reconstruction (SCR) using the long head of the biceps tendon (LHBT) autograft is a promising technique, the optimal management of its distal portion (tenotomy vs. retention) remains unclear.

**Objective:**

To compare the clinical outcomes of SCR by a long head of the biceps tendon (LHBT) autograft with biceps tenotomy or not in massive RCTs.

**Methods:**

In this retrospective cohort study, we enrolled and followed 59 patients following SCR using the LHBT between 2016 and 2021. Patients were divided into two groups based on intraoperative management of the distal LHBT: the LHBT‐retained group and the LHBT‐tenotomy group. Statistical comparisons included repeated‐measures ANOVA, two‐way mixed‐design ANOVA, chi‐square/Fisher's exact tests, and Mann–Whitney *U* tests as appropriate. The visual analog scale (VAS), American Shoulder and Elbow Surgeons (ASES) score, constant score and range of motion (ROM), and the acromiohumeral distance (AHD) were assessed as outcome measures.

**Results:**

No major surgical complications were observed in any patient after surgery. The VAS score (7.0 vs. 0.6), AHD (3.2 ± 1.1 vs. 7.8 ± 0.8 mm), ASES (38 vs. 92), constant score (41 vs. 80), and ROM were statistically improved compared to their preoperative values. All patients were further subdivided into two groups according to the management of the distal end of the LHBT after transposition and fixation (retained group: the distal part of the LHBT was retained; tenotomy group: the distal part of the LHBT was resected). The two groups had comparable baseline demographic and clinical characteristics. We found that tenotomy group showed more significant function improvement within 12 months postoperatively (*p* < 0.05) compared with retained group. Nevertheless, compared with tenotomy group, the AHD of retsained group increased by 1.9 mm (5.0 ± 1.2 mm vs. 3.1 ± 0.8mm). Postoperative imaging assessment at 2 years revealed low and comparable retear rates (grades IV–V) between groups (retained group: 9.7% vs. tenotomy group: 7.1%).

**Conclusion:**

SCR using the LHBT autograft significantly improves outcomes in massive RCTs. While both techniques are effective, patients with distal biceps tenotomy (tenotomy group) exhibited superior early functional recovery (within 12 months), whereas those with an intact distal LHBT (retained group) demonstrated significantly greater improvement in AHD. Both groups achieved comparably low retear rates.

**Level of Evidence:**

Level 4.

## Graphical abstract

SCR using LHBT is effective. Tenotomy favors early function; retention improves AHD more. Both are viable with low retear rates.

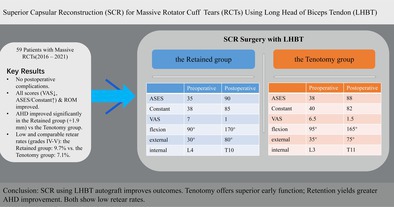

**Abbreviations**
AHD: acromiohumeral distance
ANOVA: analysis of variance
ASES: American Shoulder and Elbow Surgeons
GT: greater tuberosity
LHBT: long head of the biceps tendon
RCTs: rotator cuff tears
ROM: range of motion
SCR: superior capsular reconstruction
TFL: tensor fascia latae
VAS: visual analog scale

## 1 Introduction

Massive rotator cuff tears (RCTs) can cause significant morbidity for patients, manifesting as severe pain and functional impairment. Even with advanced techniques like tendon transfer, balloon arthroplasty, and graft bridging enhancement, successful repair remains challenging due to high failure rates and unpredictable functional outcomes. According to reports, the revision rate of repaired shoulder sleeves ranges between 9% and 94% [1, 2, 3, 4]. The integrity of the superior capsule, a critical static stabilizer of the glenohumeral joint, is invariably compromised in massive RCTs, exacerbating proximal humeral migration and dysfunction.

Superior capsule reconstruction (SCR) has been established as an effective procedure to address this pathology by reconstructing the superior capsule, thereby restoring stability and improving clinical outcomes [5, 6]. Extensive cadaveric biomechanical studies have consistently highlighted the critical role of SCR in maintaining shoulder stability and optimizing functional performance [7, 8, 9, 10]. The success of SCR is inherently linked to the selection of graft material, which has been a subject of ongoing debate. Initial techniques utilized tensor fascia latae (TFL) autografts, but these are associated with donor site morbidity and prolonged operative times. As an alternative, human dermal allografts gained popularity and demonstrated encouraging results [11]. However, concerns regarding their efficacy persist, including variability in graft thickness, higher rates of conversion to reverse shoulder arthroplasty, lack of significant improvement in AHD over time, and widely variable retear rates reported between 20% and 75% [12, 13, 14]. These factors contribute to the ongoing discussions regarding the optimal choice of graft material for SCR.

The search for an ideal graft has thus shifted toward locally available options. The long head of the biceps tendon (LHBT) has emerged as a promising autograft for SCR, preserving its supraglenoid insertion [15]. It offers several distinct advantages: excellent local availability, no additional procurement cost, and potentially less technical demand, streamlining the surgical process. These factors make the autologous LHBT an attractive choice for SCR. However, a key technical consideration remains unresolved: the optimal management of the tendon's distal portion after its proximal part is used for reconstruction. It is unclear whether performing a tenotomy provides superior outcomes or if retaining the distal biceps offers distinct advantages. Therefore, this study aimed to directly compare the clinical and structural outcomes of SCR using the LHBT autograft with versus without distal biceps tenotomy in patients with massive RCTs. Specifically, we sought to address the following points: (i) To compare the early (≤ 12 months) functional recovery profiles between the tenotomy and retention groups. (ii) To evaluate the differential impact on superior glenohumeral stability, as measured by the acromiohumeral distance (AHD), between the two techniques. (iii) To assess and compare the structural integrity (retear rates) of the reconstruction at mid‐term follow‐up. We hypothesized that both techniques would yield satisfactory outcomes, tenotomy would facilitate superior early functional recovery, whereas retention would provide greater improvement in superior stability.

## 2 Methods

### 2.1 Study Design

A retrospective evaluation was conducted on cases following massive RCT repairs from January 2016 to December 2021. In order to explore the effect of SCR using LHBT with its distal part tenotomy or not, we further compared the clinical outcomes between the retained group and tenotomy group. A retrospective protocol was established and approved by the institutional review board of Huashan Hospital, Fudan University (Approval No. 2026‐001). The primary statistical comparisons focused on intergroup analyses (retained group vs. tenotomy group) over time and preoperative versus postoperative improvements, with detailed methodologies provided in the Statistical Analysis subsection.

Patients who underwent arthroscopic SCR using the LHBT autograft for massive RCTs were included if they met the following criteria: (1) > 18 years; (2) preoperative imaging examination shows anterosuperior or posterosuperior massive RCTs (defined a tear with a diameter of 5 cm or more as described by Cofield [16] or as a complete tear of two or more tendons as described by Gerber et al. [17]); (3) serious retraction of the supraspinatus tendon to the glenoid edge or medial to it (Patte Grade III) was confirmed with preoperative imaging assessment and intraoperatively; (4) significant degeneration of the torn rotator cuff tendons (Hamada Grade 2–3, Goutallier Grade 3–4); and (5) the intra‐articular LHBT must be intact with good quality, or exhibit only mild wear and degeneration.

The exclusion criteria were as follows: (1) Preoperative imaging examination shows severe glenohumeral osteoarthritis or RCT arthropathy (Hamada grade ≥ 4); (2) arthroscopic exploration revealed LHBT dislocation, tear of intra‐articular LHBT exceeding 50% of tendon width or severe degeneration (tendon thickening with obvious longitudinal tear); (3) rheumatic or rheumatoid arthritis; (4) pigmented villonodular synovitis; (5) combined with acromioclavicular or shoulder joint dislocation; (6) combination with SLAP lesions; (7) massive RCT undergoing revision; (8) previous shoulder surgery or joint infection; and (9) paralysis of the axillary nerve or malfunction of the deltoid muscle.

### 2.2 Surgical Technique

**FIGURE 1:**
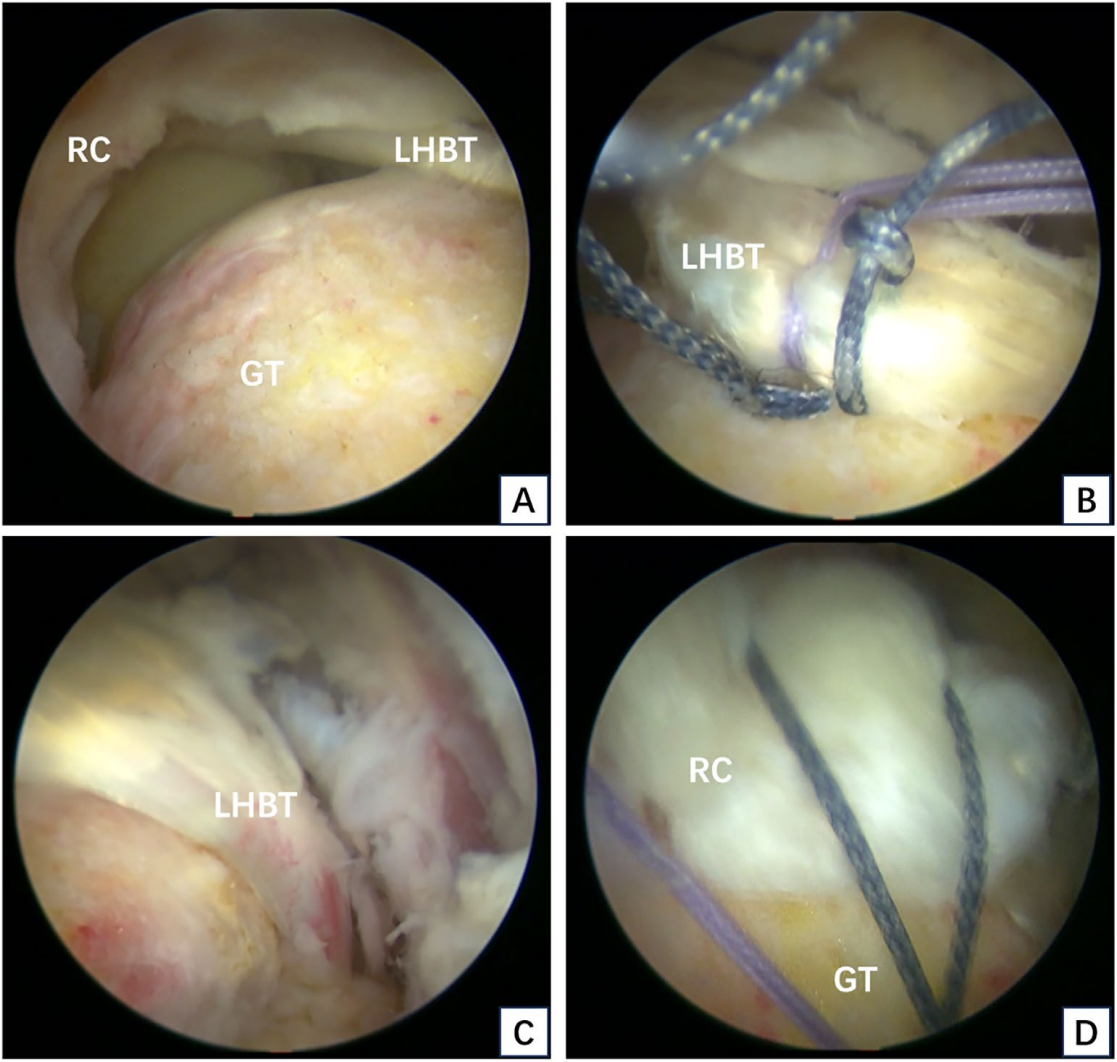
Surgical technique diagrams of retained group. (A) Massive rotator cuff tear. (B) Fixation of the transposed LHBT to the footprint. (C) Retention of an intact distal LHBT. (D) Completion of the rotator cuff repair. GT, greater tuberosity; LHBT, long head of biceps tendon; RC, rotator cuff.

**FIGURE 2:**
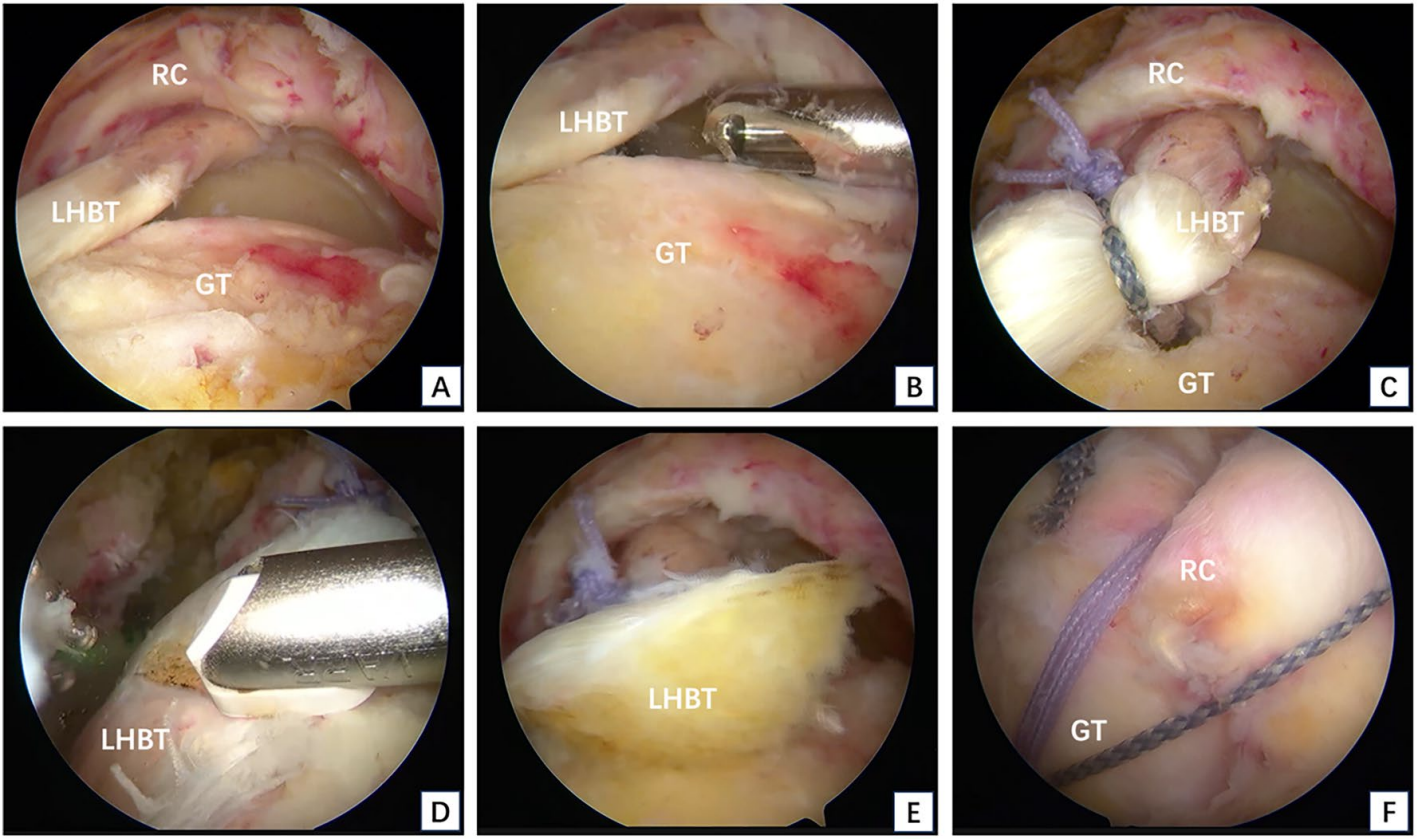
Surgical technique diagrams of tenotomy group. (A) Massive rotator cuff tear. (B) Preparation of the greater tuberosity footprint. (C) Fixation of the transposed LHBT to the footprint. (D) Tenotomy of a degenerative distal LHBT. (E) The resulting stump of the LHBT. (F) Completion of the rotator cuff repair. GT, greater tuberosity; LHBT, long head of biceps tendon; RC, rotator cuff.

The surgical technique was performed according to previous report [15, 18]. In brief, patients were positioned in the lateral decubitus position on the healthy side under general anesthesia. Standard and accessory arthroscopic portals were established as needed. A 30° arthroscope was introduced into the glenohumeral joint. A thorough evaluation was conducted to confirm the RCT extent, any associated injuries, and LHBT quality. Capsulodesis was performed for patients with joint adhesion. For those with subscapularis tendon tears, repair was performed either intra‐articularly or in the subacromial space according to the tear size. Next, lateral and anterolateral portals were established, and extensive bursectomy was performed. Acromioplasty was performed when necessary to preserve the coracoacromial ligament. Finally, the rotator cuff repair (RCT) was started (Figures 1 and 2).

1. First, the tear size and retraction of the rotator cuff were evaluated. Massive rotator cuff with the retracted supraspinatus tendon was confirmed arthroscopically. If it was still difficult to mobilize the torn rotator cuff back to its footprint after release, if this could only be achieved with high tension, or if no significant LHBT tear or degeneration was detected, the superior capsular reconstruction (SCR) was conducted with the LHBT.
2. The greater tuberosity (GT) was prepared using a motorized bur. The LHBT was then translocated to the footprint area of the supraspinatus tendon near the articular surface under appropriate tension. Usually, rerouting posteriorly about 1.5 cm from the footprint, a three‐wire Healix anchor (Mitek) with a diameter of 5.5 mm was implanted, and the LHBT was fixed with Lasso technique.
3. Tendon tension and tear shape were assessed, and 1–2 Healix anchors (Mitek) with a diameter of 4.5 mm were implanted posterior to the fixation site of the LHBT to suture the infraspinatus tendon. Supraspinatus tendon repair was performed partially or completely according to the tension. The same anchors used to fix the LHBT could also be used to repair the supraspinatus tendon.
4. The repaired supraspinatus and infraspinatus tendons were gathered toward the LHBT. Side‐by‐side repair was performed based on the tension; however, the infraspinatus tendon was not repaired side by side with the LHBT.
5. After the repair was completed, the decision to resect or retain the distal LHBT was based on tendon integrity. Retained group: If the distal LHBT was intact with no obvious tear or degeneration, it was retained. Tenotomy group: If the distal part of the LHBT was torn by more than 50% of its width or showed severe degeneration (e.g., obvious longitudinal tear), a tenotomy was performed. After the operation, hemostasis was thoroughly carried out, and the skin incision was sutured.

### 2.3 Postoperative Rehabilitation

Sports medicine‐focused rehabilitation specialists provided organized functional exercise program assistance to all patients. After surgery, the affected arm was positioned in an abduction pillow set at 30° for a period of 6 weeks to ensure proper recovery. Active range of motion (ROM) exercises for the elbow, wrist, and hand, as well as arm muscle strengthening, scapular stability exercises, and shoulder muscle relaxation training were started 1 day after surgery. Besides, passive shoulder mobilization (forward flexion < 90° and external rotation) commenced. Physiotherapy and ice were used to reduce swelling and inflammation. Strengthening exercises were introduced 3 months postoperatively with a gradual progression in intensity. Resistance exercises were advised starting 6 months after surgery to further enhance functional recovery.

### 2.4 Patient Evaluation

The following information was collected before surgery and at 3, 6, 12, and 24 months after surgery: age, gender, body mass index, RCT type, preoperative motion range, muscle fatty infiltration (Goutallier classification), rotator cuff retraction (Patte classification), presence of acromial spurs, glenohumeral joint degeneration (Hamada–Fukuda classification), tear size in the anteroposterior aspect, AHD, American Shoulder and Elbow Surgeons (ASES) score, visual analog scale (VAS), and constant score. AHD was measured on standardized anteroposterior shoulder radiographs taken with the arm in neutral rotation. Two independent observers evaluated the final ROM and gathered outcome metrics.

Tendon healing status was assessed using ultrasound or magnetic resonance imaging (MRI). Based on the classification system described by Sugaya et al. and validated for ultrasound ultrasound by Barth et al. [19], lower grades (I‐III) indicate intact or partially healed tendons, and higer grades (IV‐V) indicate structural failure. Structural failure was defined as a retear of the repaired rotator cuff tendons (supraspinatus and/or infraspinatus), classified as higher grades (IV or V) on postoperative imaging.

### 2.5 Statistical Analysis

Continuous data were noted as means and standard deviations, while categorical data were presented as percentages. Two researchers independently measured those samples without conferring. The resulting inter‐rater reliability coefficients (Cohen's *κ* ≈ 0.85 and ICC ≈ 0.87) were high. Regarding statistical comparisons, continuous variables were analyzed using repeated measures ANOVA for preoperative vs. postoperative comparisons and a two‐way mixed‐design ANOVA for intergroup analyses. Categorical variables were compared using chi‐square or Fisher's exact tests. Non‐parametric data were assessed with the Mann–Whitney *U* test. These analyses were described in the Statistical Analysis subsection. The SPSS 25.0 software (SPSS Inc., Chicago, IL) was applied in this research. A *p* value below 0.05 was deemed statistically significant. Prospective power analysis indicated 50 participants provided 80% power to detect target effect sizes.

## 3 Results

### 3.1 Patient Demographics and Overall Outcomes

**FIGURE 3:**
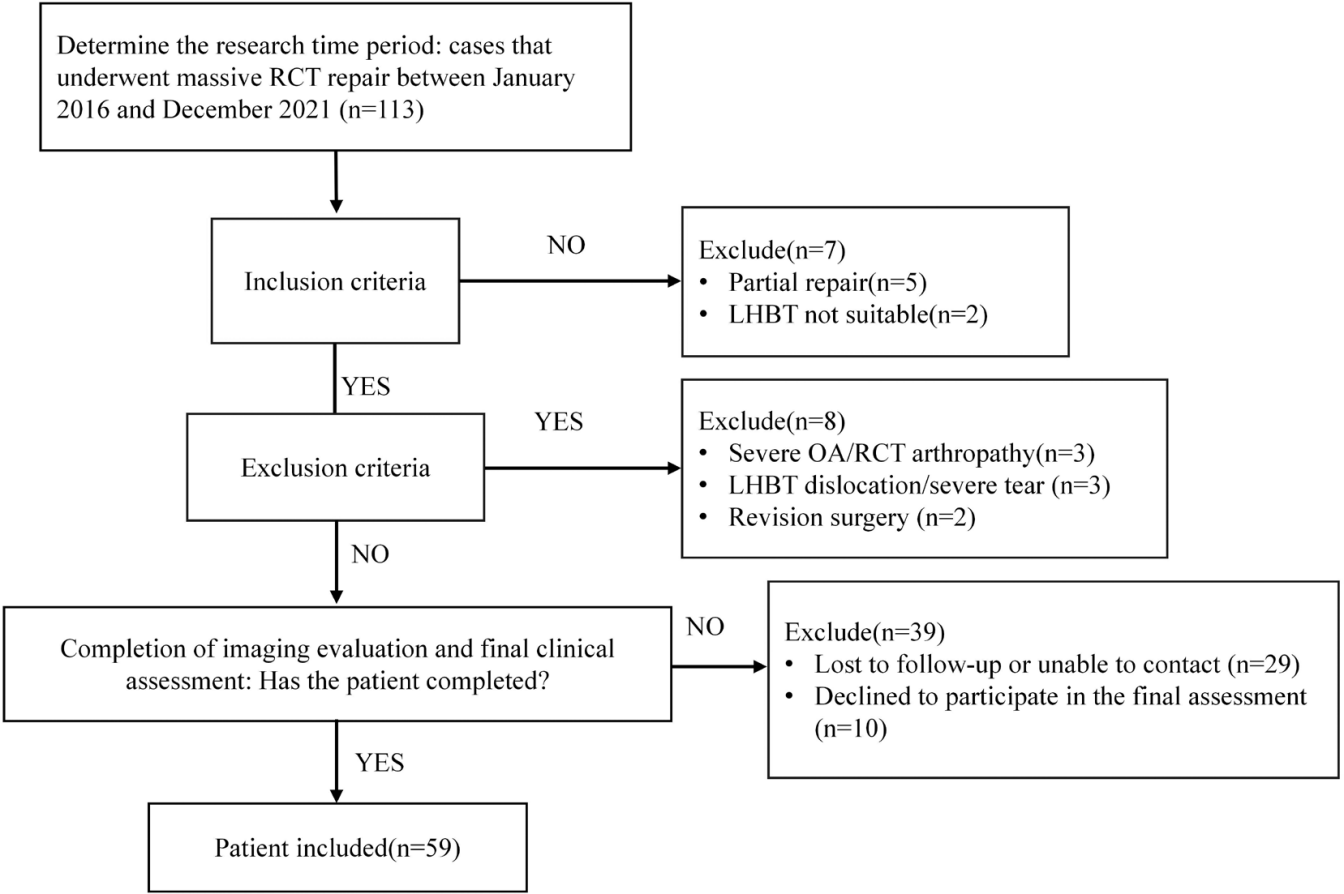
A flowchart of patient selection.

**FIGURE 4:**
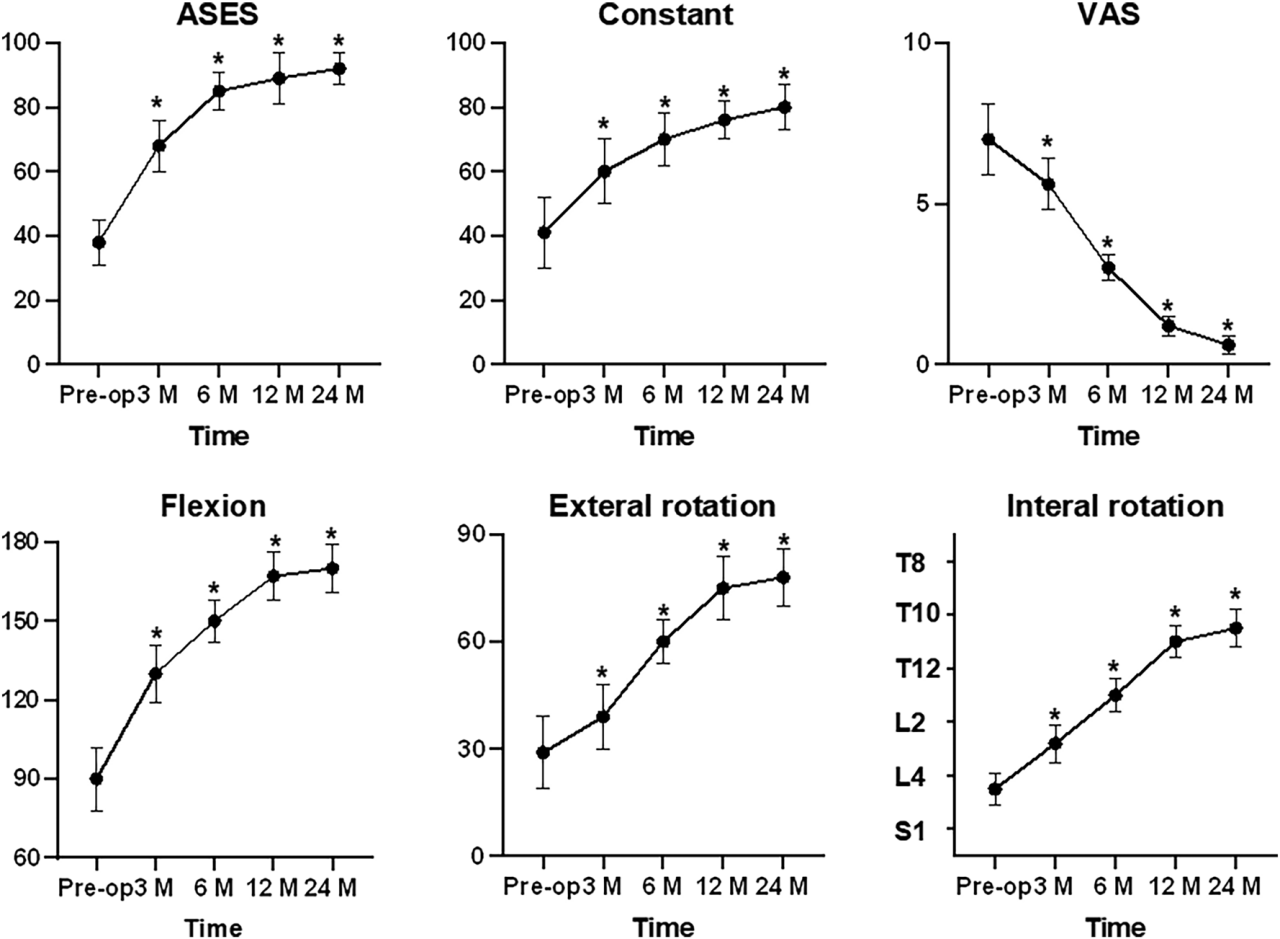
Comparison of clinical outcomes between preoperative and postoperative functional outcomes. ASES, American Shoulder and Elbow Surgeons; VAS, visual analog scale. *p < 0.05.

Among 113 consecutive patients who underwent surgery for massive RCTs between 2016 and 2021, only 59 patients fulfilled the requirements for inclusion and finished both imaging evaluation and final clinical assessments (Figure 3). The AHD significantly increased from 3.2 ± 1.1 to 7.8 ± 0.8 mm (*p* = 0.002). As shown in Figure 4, during the final follow‐up, the VAS score significantly decreased from 7.0 to 0.6 (*p* = 0.011), ASES score from 38 to 92 (*p* = 0.001), and constant score from 41 to 80 (*p* = 0.007) in all patients. In addition, active ROM was significantly improved (flexion from 92° to 167°, external rotation from 29° to 75°, and internal rotation from L3 to T10) at the final follow‐up compared with preoperative results in all patients. Furthermore, after SCR using LHBT without distal tenotomy, the AHD increased by 1.9 mm in the retained group (5.0 ± 1.2 vs. 3.1 ± 0.8 mm; *p* < 0.05).

Patient characteristics are presented in Table 1. The two groups had comparable data like follow‐up duration and RCT methods. Their preoperative evaluations, such as Acromial spurs classification, AHD, Hamada–Fukuda classification, supraspinatus retraction, tear size, VAS for pain, ASES score, and constant score, did not show a significant difference.

**TABLE 1:** Baseline features.

	Retained group	Tenotomy group	*p*
Numbers	31	28	0.61
Age (years)	62.8 ± 5.7	65.3 ± 4.6	0.78
Male/female	12/19	9/19	0.56
Body mass index	26.7 ± 3.2	25.1 ± 3.9	0.72
Diabetes	6	7	0.81
Smoking	11	9	0.73
Hyperlipidemia	8	6	0.69
Follow‐up duration	26.3 ± 1.3	27.4 ± 2.2	0.76
Rotator cuff repair methods, *n*			
Single‐row	11	9	0.93
Suture bridge	20	19	0.86
Preoperative x‐ray			
Acromial spurs classification I/II/III, *n*	11/12/8	10/9/9	0.36
AHD (mm)	5.7 ± 1.9	6.4 ± 1.6	0.69
Hamada–Fukuda classification I/II/III, *n*	0/21/10	0/17/11	0.81
Preoperative MRI, *n*			
Supraspinatus retraction Patte classification II/III	16/15	13/15	0.59
Fatty infiltration grade (3/4)			
Supraspinatus	22/9	18/10	0.83
Infraspinatus	24/7	19/9	0.75
Tear size in anteroposterior aspect (cm)	5.5 ± 1.2	5.8 ± 1.4	0.66
VAS	7.6 ± 0.9	6.8 ± 1.1	0.68
ASES	35 ± 11	40 ± 10	0.85
Constant score	38 ± 9	42 ± 10	0.69

*Note:* This table shows the patient characteristics between the Retained group (LHBT retained) and the Tenotomy group (LHBT with distal tenotomy).

Abbreviations: AHD, acromiohumeral distance; ASES, American Shoulder and Elbow Surgeons; VAS, visual analog scale.

### 3.2 Comparison Between the Retained and Tenotomy Groups

**FIGURE 5:**
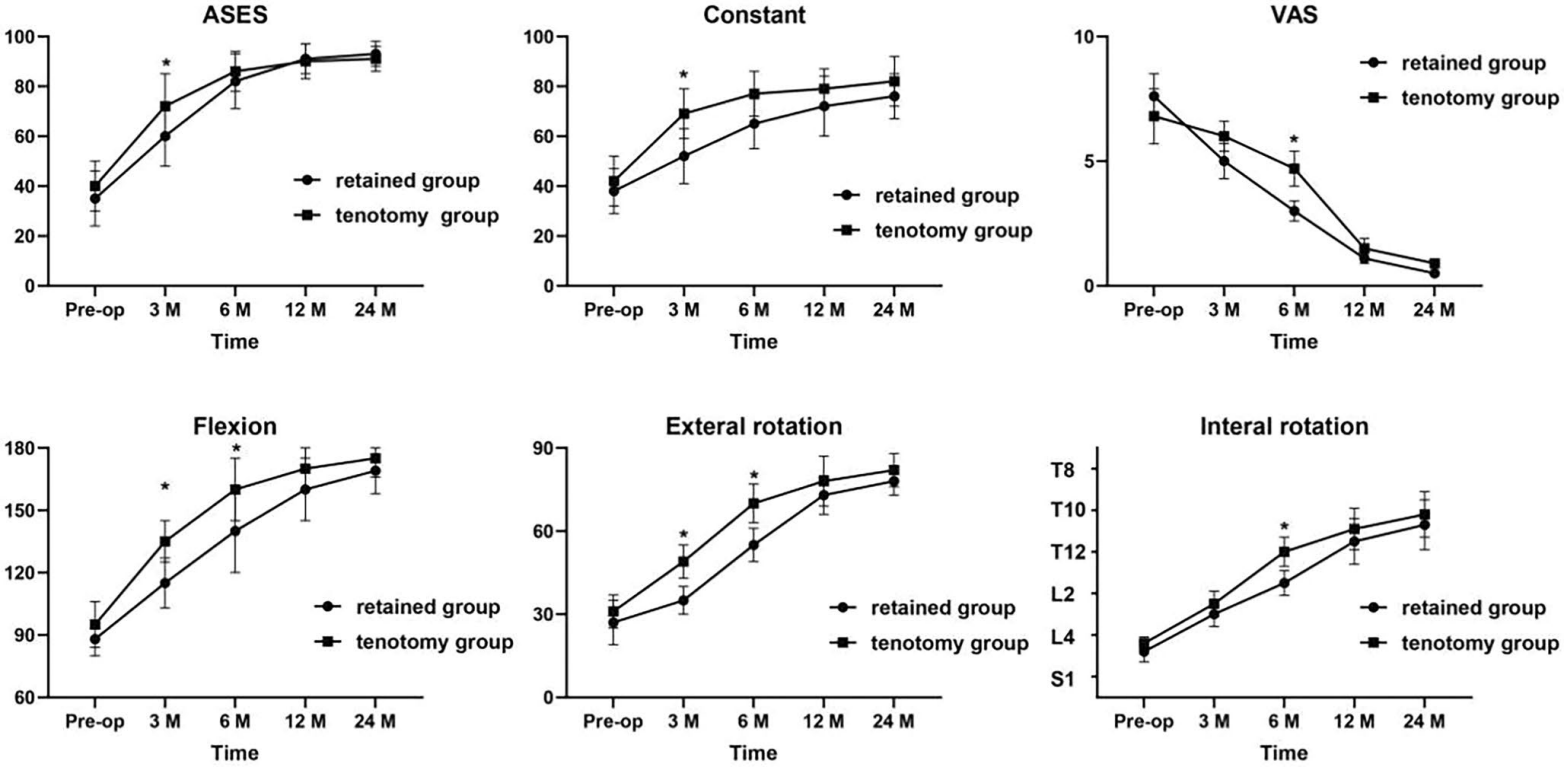
Clinical outcomes. ASES, American Shoulder and Elbow Surgeons; VAS, visual analog scale. *p < 0.05.

As illustrated in Figure 5, the final functional outcomes were comparable between the study groups. At the 3‐, 6‐, 12‐, and 24‐month postoperative follow‐ups, both groups showed a continuous increase in ASES scores (retained group: 35–90; tenotomy group: 38–88) and constant scores (retained group: 38–85; tenotomy group: 40–82), along with a gradual decrease in VAS scores (retained group: 7 to 1; tenotomy group: 6.5 to 1.5). Significant intergroup differences were observed at some time points marked with *p < 0.05. In terms of joint ROM, preoperatively, the levels of flexion, external rotation, and internal rotation were comparable between the two groups. Over time postoperatively, the flexion angles (retained group: 90°–170°; tenotomy group: 95°–165°) and external rotation angles (retained group: 30°–80°; tenotomy group: 35°–75°) showed a continuous increase, while the internal rotation levels (retained group: L4 to T10; tenotomy group: L3 to T11) improved significantly. Statistically significant intergroup differences were noted at some time points (*p* < 0.05), reflecting the differing time‐dependent efficacy profiles of the two intervention methods.

The differential recovery patterns between the two groups are visually exemplified in Figures S1 and S2, which provide representative imaging photographs of one typical case from the retained group and one from the tenotomy group at preoperative and key postoperative timepoints.

### 3.3 Structural Outcomes (Imaging Assessment)

**FIGURE 6:**
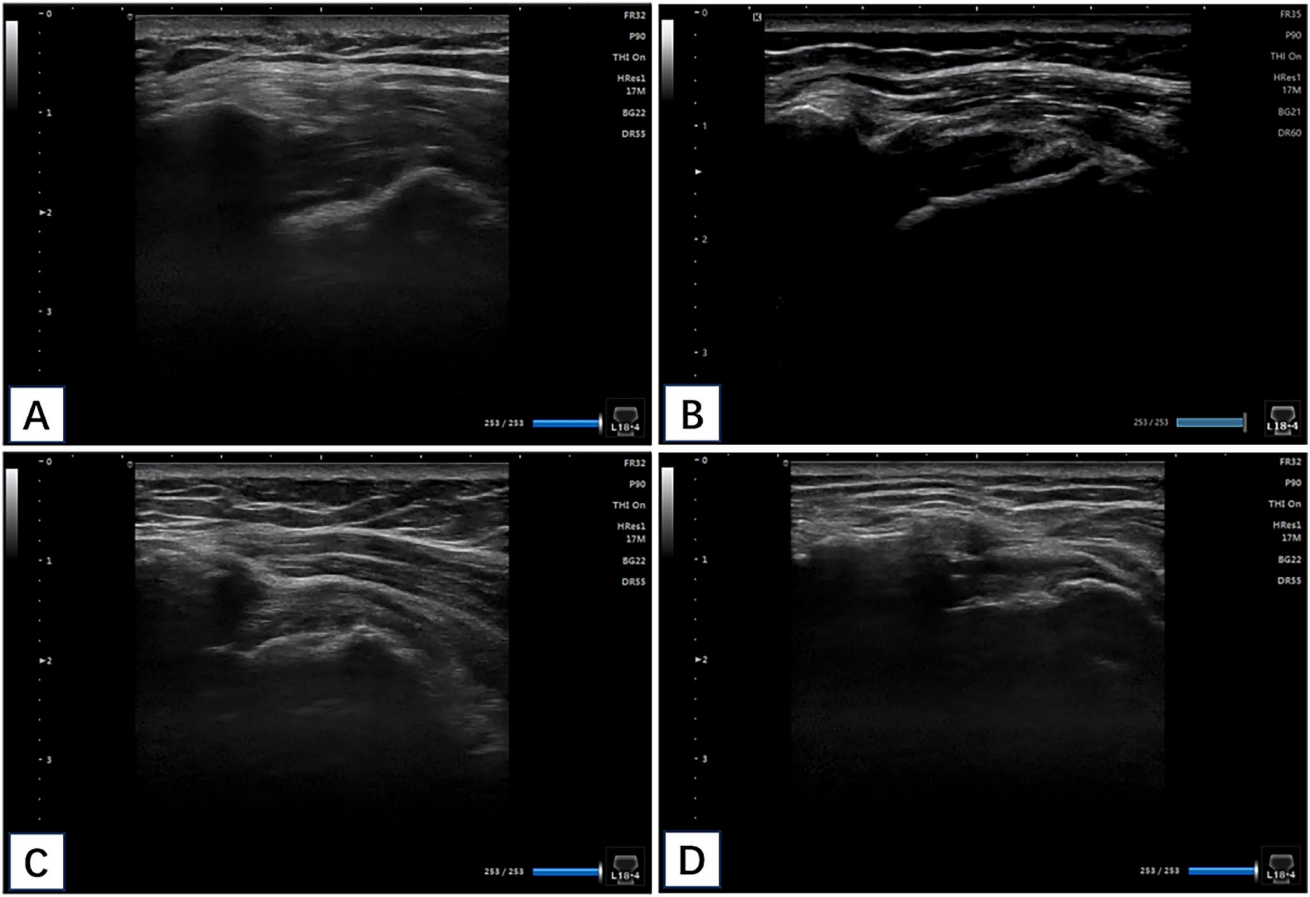
Postoperative ultrasound imaging. (A) Retained group, Grade I: Intact tendon repair with sufficient thickness. Clinical outcome: ASES = 90, VAS = 1. (B) Retained group, Grade IV: Focal full‐thickness tear indicating structural failure. Clinical outcome: ASES = 75, VAS = 3. (C) Tenotomy group, Grade I: Intact tendon repair with sufficient thickness and homogeneous low echogenicity. Clinical outcome: ASES = 95, VAS = 1. (D) Tenotomy group, Grade IV: Small, focal full‐thickness tear. Clinical outcome: ASES = 85, VAS = 2. ASES, American Shoulder and Elbow Surgeons; VAS, visual analog scale.

As shown in Table 2, imaging assessment at 2 years after surgery revealed that the repaired rotator cuff tendon was still intact (grades I–III) in 28 cases (90.3%) of the retained group and 26 patients (92.9%) of the tenotomy group. Representative ultrasound images illustrating these healing characteristics are presented in Figure 6. The figure provides visual examples of both excellent tendon integrity (grade I) and structural failure (grade IV) from each surgical group, alongside their corresponding clinical outcome scores, offering a direct correlation between anatomical healing and functional results.

**TABLE 2:** Repaired cuff integrity at 2 years postoperatively by imaging grade.

	Retained group	Tenotomy group	*p*
Grade I	12	10	0.71
Grade II	11	9	0.59
Grade III	5	7	0.86
Grade IV	2	1	0.63
Grade V	1	1	0.81

*Note:* The table shows the repaired cuff integrity at 2 years postoperatively according to Sugaya classification, comparing the Retained group (LHBT retained) and the Tenotomy group (LHBT with distal tenotomy).

Table 3 showed that postoperative functional outcomes were significantly worse in cases with structural failure (retears of the repaired rotator cuff tendons) compared to those with intact repairs. These outcomes remained superior to the preoperative conditions.

**TABLE 3:** Postoperative functional outcomes in cases having intact and retear of tendon.

	Intact tendon (*n* = 54)	Retear tendon^a^ (*n* = 5)	*p*
VAS	0.7 ± 0.5	1.2 ± 0.8	0.31
ASES	89 ± 6	76 ± 8	0.02
Constant score	82 ± 7	71 ± 9	0.03
Flexion (°)	165 ± 11	151 ± 8	0.03
External rotation (°)	78 ± 10	69 ± 8	0.28
Internal rotation	T10	T8	0.01

*Note:* The table shows the postoperative functional outcomes in cases having intact and retear tendon. Retear tendon^a^ refers to the supraspinatus or the infraspinatus tendon.

Abbreviations: ASES, American Shoulder and Elbow Surgeons; VAS, visual analog scale.

No major surgical complications (e.g., infection, neurovascular injury, or deep vein thrombosis) were observed during the follow‐up period. No patients required revision surgery, and no clinically significant Popeye deformity was reported.

## 4 Discussion

The principal finding of this comparative study is that while SCR using the LHBT autograft significantly improves outcomes irrespective of distal tendon management, the choice between tenotomy and retention results in distinct recovery patterns. Specifically, patients in the tenotomy group experienced significantly superior early functional recovery within the first 12 months, whereas patients in the retained group achieved a significantly greater increase in AHD. Both strategies, however, led to comparably low and satisfactory retear rates at 2 years.

### 4.1 Early Functional Recovery

The observed trajectory of continuous improvement over 24 months aligns with the typical recovery pattern after extensive rotator cuff surgery. The initial phase (up to 12 months) is characterized by biological healing, scar maturation at the tendon‐bone interface, and the recovery of passive and active ROM [20]. The subsequent phase (12–24 months) often involves further neuromuscular adaptation, muscle strengthening, and the patient's gaining confidence in using the limb for more demanding activities. This prolonged healing and functional adaptation period underscores the importance of long‐term follow‐up and persistent rehabilitation in achieving a final, stable outcome [21]. Within this general framework, our findings delineate two distinct recovery pathways dictated by the management of the distal LHBT.

### 4.2 Superior Stability and AHD Restoration

The superior early functional recovery observed in the tenotomy group represents a clinically relevant finding. This rapid improvement in ASES and constant scores may be attributed to the specific approach of addressing a compromised tendon. Performing a tenotomy of a degenerated or torn distal LHBT likely eliminates a potent pain generator and source of mechanical irritation. This allows the SCR construct to stabilize and function without the adverse influence of the pathological tendon, thereby facilitating accelerated early functional gains. This early improvement can positively impact patient satisfaction and engagement in rehabilitation. Conversely, the significantly greater improvement in AHD observed in the retained group suggests a different mechanism, attributable to the preservation of a healthy tendon. In these patients, the intact, tenodised LHBT is theorized to continue functioning as a dynamic stabilizer and humeral head depressor. This preserved biomechanical role likely enhances superior joint stability and centering, which is radiographically reflected in the greater increase in AHD. The trend toward better pain relief in this group may also be related to the maintained proprioceptive feedback and cushioning effect of the intact tendon. Our technique, which utilizes the LHBT autograft while preserving its supraglenoid insertion, offers theoretical advantages such as maintained blood supply and labral integrity [22, 23, 24, 25, 26, 27, 28, 29]. More importantly, our comparative results demonstrate that this graft can be utilized effectively via two distinct strategies (tenotomy vs. retention), each yielding a unique clinical profile, as outlined above.

### 4.3 Structural Integrity and Retear Rates

The degenerative LHBT is often considered a pain generator, and LHBT tenotomy has been shown to improve symptoms. Performing a tenodesis of the LHBT to the GT with a concomitant distal tenotomy enhances the RCT while simultaneously eliminating the degenerative LHBT as a potential pain generator. Therefore, our approach involves performing a tenotomy of the distal portion of the LHBT within the bicipital groove while utilizing the proximal portion for SCR. This method allows us to leverage the biomechanical benefits of the proximal LHBT graft for augmentation of RCT, while resecting the degenerative distal portion to eliminate a potential pain generator. Alpantaki et al. [30] concluded a high prevalence of sensory sympathetic fibers at the glenoid tendon origin, suggesting that using the LHBT as an autograft for SCR could induce postoperative pain. However, studies have shown no significant difference in the ROM or postoperative pain between different surgical techniques, indicating that once disconnected from the distal portion, the proximal part of the LHBT is not a pain generator and can be a local tissue autograft. A relevant consideration when performing a tenotomy is the potential for a “Popeye” deformity. In the present study, we specifically monitored for this sign during follow‐up. It is important to note that no patient in either group developed a clinically significant Popeye deformity that was reported as a concern or required further intervention. While prior literature, such as the study by MacDonald et al. which compared isolated tenotomy to tenodesis, reported a higher incidence of Popeye deformity after tenotomy (33% vs. 10%) [31], our findings within the context of SCR using the LHBT autograft suggest that the clinical relevance of this cosmetic concern may be different. We postulate that the specific surgical sequence—where the proximal LHBT is securely incorporated into the robust SCR construct and tenodised to the GT—may adequately stabilize the biceps muscle and mitigate the distal retraction that causes a prominent Popeye deformity. Therefore, while the possibility exists, it did not manifest as a clinically relevant issue in our cohort, allowing the functional benefits of tenotomy for damaged tendons to be realized without this potential drawback. This finding underscores the importance of considering functional outcomes and cosmetic concerns while selecting the optimal surgical approach.

A pivotal aspect of our study design is that the management strategy was tailored to the intraoperative condition of the distal LHBT. This protocolized approach—retaining a healthy tendon (retained group) and performing tenotomy on a compromised one (tenotomy group)—directly tailors the surgical technique to the patient's intraoperative pathology. Consequently, the outcomes differences are best interpreted as the result of applying the optimal strategy to distinct intraoperative scenarios, rather than a direct comparison of techniques in identical scenarios. This reflects a pragmatic surgical algorithm.

There were a few limitations to this study. First, there was no measurement of muscle strength recovery, which could have provided additional insight into patients' post‐surgical improvement. Second, the intra‐articular LHBT quality is crucial for this technology; however, not all patients with large to massive RCTs can get it. Third, it was a single‐center, small‐sample size study. To establish the therapeutic effect of this technique more robustly, future studies should include measurements of muscle power recovery, assessing the intra‐articular quality of the LHBT, and involve multicenter trials with larger sample sizes. Such studies would provide a more comprehensive evaluation of the technique's efficacy and generalizability. Fourth, the absence of a biceps‐specific outcome score limits our ability to capture more nuanced, tendon‐specific functional or cosmetic outcomes. Future studies would benefit from incorporating such tools to provide a more comprehensive assessment. Despite these limitations, the findings of this study offer clear guidance for clinical practice. Surgeons performing SCR with the LHBT autograft can tailor their technique based on patient‐specific goals. If the priority is to achieve the fastest possible functional recovery in the first postoperative year, performing a tenotomy of a degenerated distal tendon is advantageous. Conversely, if the primary objective is to maximize superior stability and AHD restoration, retaining an intact and healthy distal LHBT appears to be the preferable approach. The intraoperative assessment of the LHBT's quality thus becomes a critical step in this decision‐making process. When the distal LHBT is degenerated or torn, tenotomy facilitates faster early recovery. When it is intact and healthy, retention maximizes superior stability and AHD improvement. This allows for a personalized surgical strategy that leverages the unique benefits of each technique based on objective intraoperative findings.

## 5 Conclusion

SCR with the LHBT autograft is an effective treatment for massive RCTs. The management of the distal tendon dictates the recovery pattern: tenotomy facilitates superior early functional recovery, whereas retention provides significantly greater improvement in superior stability, as measured by AHD. Both techniques achieve equally satisfactory and low retear rates, allowing surgeons to individualize treatment based on intraoperative findings and patient expectations.

## Author Contributions

**Jiani Chen:** investigation, data curation, validation, supervision, writing – review and editing. **Yimeng Yang:** writing – original draft, software, resources. **Shurong Zhang:** investigation, formal analysis. **Yang Wu:** methodology. **Jiwu Chen:** project administration. **Shiyi Chen:** data curation, visualization. **Xiliang Shang:** writing – review and editing, conceptualization, funding acquisition.

## Funding

This work was supported by the National Natural Science Foundation of China (Grant Nos. 81972125, 82172510).

## Ethics Statement

The research related to human use has been conducted in accordance with all the relevant national regulations and institutional policies and the tenets of the Helsinki Declaration, and it has been approved by the institutional review board of Huashan Hospital, Fudan University (Approval No. 2026‐001). For this retrospective cohort study, informed consent was obtained from all individuals for the use of their clinical data and participation in postoperative follow‐up assessments.

## Conflicts of Interest

The authors declare no conflicts of interest.

## Supporting information


**Figure S1:** Representative radiographic findings in the retained and tenotomy groups.
**Figure S2:** Representative magnetic resonance imaging (MRI) findings in the retained and tenotomy groups.

## Data Availability

The data that support the findings of this study are available on request from the corresponding author. The data are not publicly available due to privacy or ethical restrictions.
