# Supplementary material for: Clinical Effect of Superior Capsular Reconstruction With Long Head of the Biceps Tendon Autograft: Biceps Tenotomy Versus Retention in Massive Rotator Cuff Tears
Source: Orthop Surg. 2026 Mar 17;18(4):710–20. doi: 10.1111/os.70278 (PMC13056468; doi:10.1111/os.70278)
Supplement: Supplementary file 1 — Figure S1: Representative radiographic findings in the retained and tenotomy groups. Figure S2: Representative magnetic resonance imaging (MRI) findings in the retained and tenotomy groups. [file OS-18-710-s001.docx]

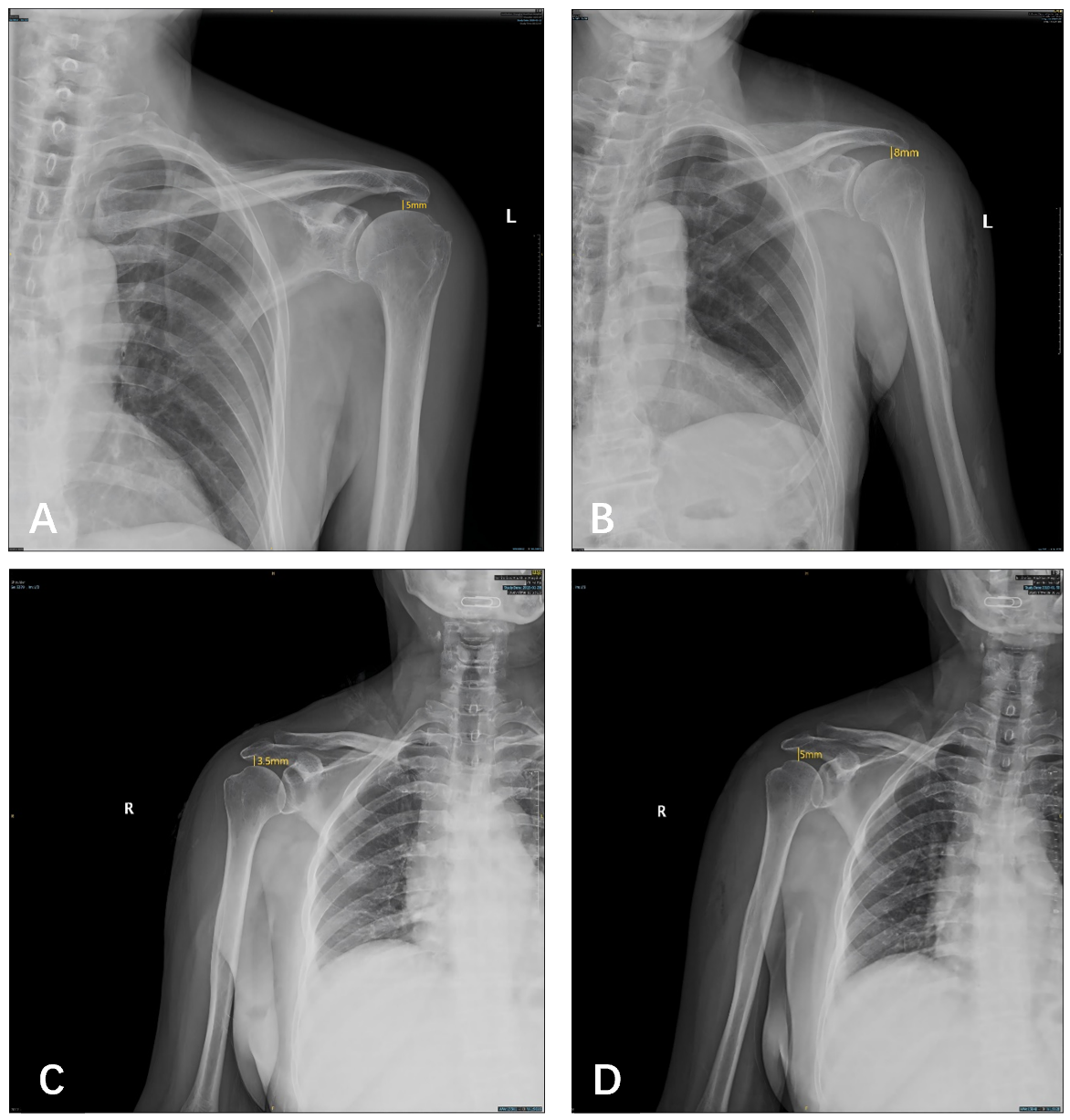


**Figure S1. Representative radiographic findings in the Retained and Tenotomy groups.**

**(A) Retained group, Preoperative:** Anteroposterior radiograph of the shoulder shows superior migration of the humeral head. The acromiohumeral distance (AHD) measures 5.0 mm (between yellow short lines).

**(B) Retained group, Postoperative:** Radiograph of the same patient demonstrates restored glenohumeral alignment with a significantly improved AHD of 8.0 mm (between yellow short lines).

**(C) Tenotomy group, Preoperative:** Anteroposterior radiograph shows superior humeral head migration (AHD: 3.5 mm).

**(D) Tenotomy group, Postoperative:** Radiograph of the same patient shows improvement in humeral head position (AHD: 5.0 mm).

**
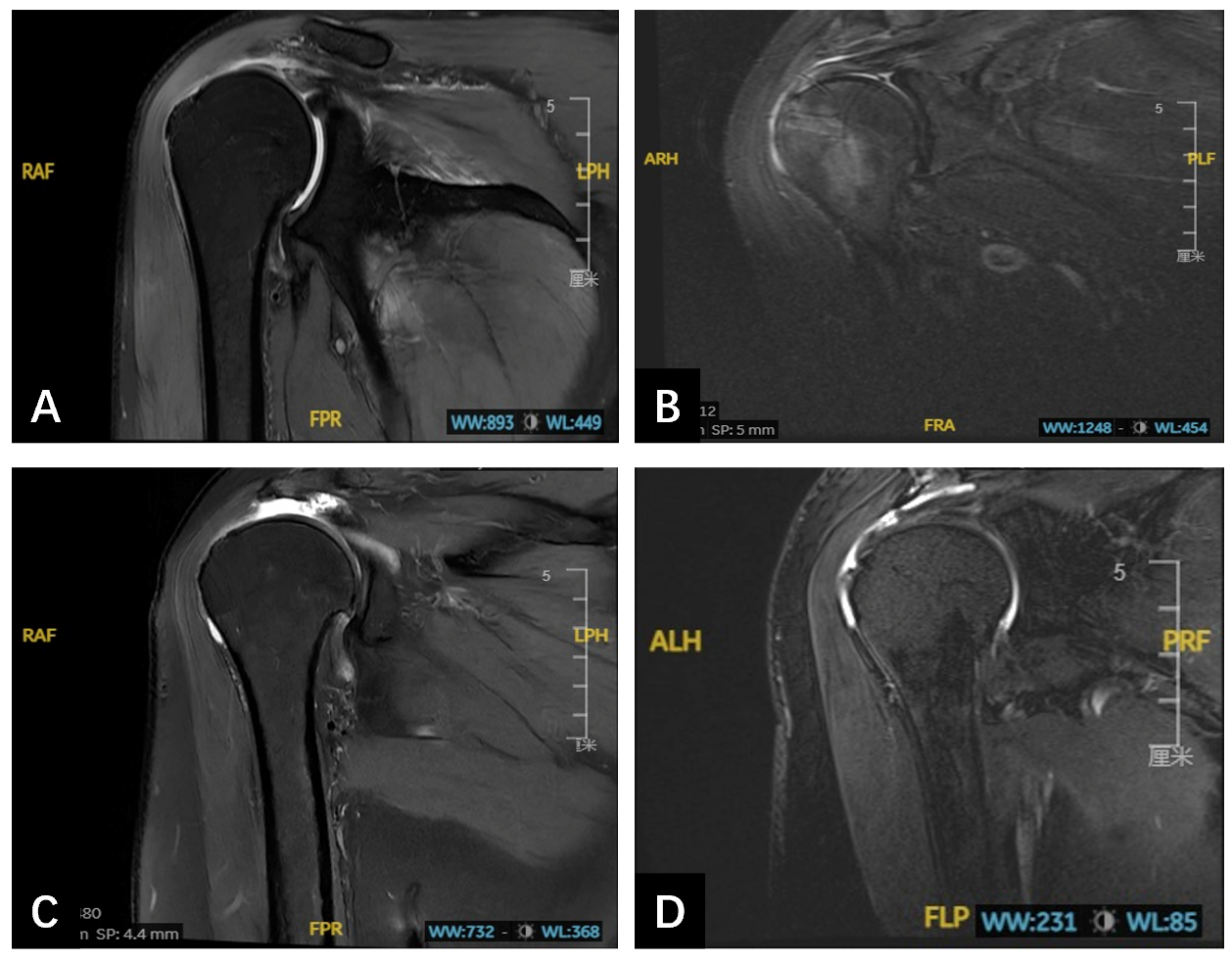
Figure S2. Representative magnetic resonance imaging (MRI) findings in the Retained and Tenotomy groups.**

**(A) Retained group, Preoperative:** MRI demonstrates a massive, retracted full-thickness tear of the supraspinatus tendon.

**(B) Retained group, Postoperative:** MRI shows a healed, low-signal-intensity tendon at the footprint, indicating successful rotator cuff repair and superior capsular reconstruction.

**(C) Tenotomy group, Preoperative:** MRI shows a massive rotator cuff tear with tendon retraction.

**(D) Tenotomy group, Postoperative:** MRI demonstrates a healed, continuous low-signal tendon at the greater tuberosity, confirming structural integrity.
